# Airborne Particulate Matter and Acute Lung Inflammation: Strak et al. Respond

**DOI:** 10.1289/ehp.1205860R

**Published:** 2013-01-01

**Authors:** Maciej Strak, Nicole A.H. Janssen, Ilse Gosens, Flemming R. Cassee, Erik Lebret, Krystal J. Godri, Ian S. Mudway, Frank J. Kelly, Roy M. Harrison, Bert Brunekreef, Maaike Steenhof, Gerard Hoek

**Affiliations:** 1 National Institute for Public Health and the Environment (RIVM), Bilthoven, Netherlands, E-mail: m.m.strak@uu.nl; 2 MRC-HPA Centre for Environmental Health, King’s College London, London, United Kingdom; 3 Division of Environmental Health and Risk Management, University of Birmingham, Birmingham, United Kingdom; 4 Institute for Risk Assessment Sciences Utrecht University, Utrecht, Netherlands

We thank Gangamma for a number of excellent observations and would like to respond to the issues raised.

Gangamma points out that the levels of fractional exhaled nitric oxide (FE_NO_) may be influenced by the site of particle deposition in the lung and requests further explanation on how this could affect the analysis and conclusion of our study (Strak et al. 2012). In an observational study such as ours, it is not possible to assess precise locations of particle deposition in the respiratory tract. Although variations in location of particle deposition likely introduced some noise in the FE_NO_ readings, we could not take this into account in the regression model.

Gangamma notes that many components of particulate matter (PM) can induce neutrophil inflammation in the lung; thus, focusing only on FE_NO_ may not sufficiently reflect their effects. FE_NO_ is an indicator of airway inflammation that is used fairly often in observational and experimental studies. As we stated above, in a study such as ours (Strak et al. 2012), it would be very challenging to address many possible inflammation pathways. In addition, the focus of our study was more on the components and characteristics of air pollution and associated health effects. We included other inflammatory markers (e.g., interleukin-6, neutrophils) measured both in blood and nasal lavage in our health measurements, but those were outside of the scope of our paper.

The next issue raised by Gangamma deals with the suggestion that many of the measured FE_NO_ values could be within error range of the measurement instrument; therefore, data on the precision of the measurements should be provided and explanation should be given on how it could affect the regression analysis. Measurement error is an issue in every observational, as well as experimental, study. It reduces the chance of finding associations with external variables (also subject to measurement error); however, in a regression analysis, measurement error in the dependent variable does not introduce bias in the size of the regression coefficient.

Gangamma’s last comment concerned the suitability of using the CPC 3007 portable condensation particle counter (TSI, St. Paul, MN) for ambient measurements. We used the CPC 3007 only to characterize the air pollution inside the van during transport of the participants to and from the sampling locations. Measurements at the sampling locations were performed using a CPC 3022a (TSI), which is an instrument commonly used in similar experimental studies for characterization of particle number concentration in ambient air.
